# Supplementary material for: Junior to senior transition of male elite junior tennis players: A retrospective study
Source: PLoS One. 2024 Oct 23;19(10):e0309472. doi: 10.1371/journal.pone.0309472 (PMC11498654; doi:10.1371/journal.pone.0309472)
Supplement: S1 File — (PDF) [file pone.0309472.s001.pdf]

no	Year of the tournament	Nomination	Continent	Qi	WJTF results	ATP status
1	2012	3	4	1	4	0
2	2012	3	1	1	4	0
3	2012	2	1	3	4	0
4	2012	1	1	2	4	0
5	2012	2	4	2	1	0
6	2012	2	5	2	4	0
7	2012	1	4	4	2	0
8	2012	3	6	1	3	0
9	2013	3	1	3	4	0
10	2013	3	2	4	3	0
11	2013	3	2	3	4	0
12	2013	2	4	2	2	0
13	2013	1	3	3	1	0
14	2014	2	5	1	2	0
15	2014	1	5	4	2	0
16	2014	3	2	1	3	0
17	2014	1	2	1	3	0
18	2014	3	4	2	4	0
19	2014	3	6	1	3	0
20	2014	2	2	2	4	0
21	2014	1	2	1	4	0
22	2014	1	6	1	1	0
23	2014	2	6	1	2	0
24	2015	3	6	1	4	0
25	2015	1	6	2	4	0
26	2015	3	2	1	1	0
27	2015	3	1	2	4	0
28	2015	2	1	1	4	0
29	2015	2	4	1	3	0
30	2015	3	2	2	2	0
31	2015	2	5	2	1	0
32	2016	3	5	1	1	0
33	2016	3	6	1	2	0
34	2016	1	6	2	2	0
35	2016	3	1	2	4	0
36	2016	2	1	3	4	0
37	2016	2	2	1	3	0
38	2016	3	5	4	4	0
39	2016	3	2	1	1	0
40	2016	1	2	1	1	0
41	2016	3	2	1	4	0
42	2016	1	4	2	3	0
43	2016	2	6	1	4	0
44	2016	2	4	2	2	0
45	2016	3	6	1	1	0
46	2012	3	6	2	1	1
47	2013	2	2	1	4	1
48	2013	3	4	3	4	1
49	2015	1	1	1	4	1

50	2013	2	4	4	4	1
51	2015	2	4	1	4	1
52	2014	2	4	1	2	1
53	2013	2	1	2	4	1
54	2016	1	4	4	3	1
55	2012	2	6	2	1	1
56	2013	1	4	1	4	1
57	2014	2	6	1	3	1
58	2012	1	6	1	2	1
59	2015	3	5	2	1	1
60	2012	2	4	3	2	1
61	2015	1	5	1	1	1
62	2013	2	2	1	3	1
63	2013	2	4	1	1	1
64	2014	3	1	1	4	1
65	2015	1	1	1	3	1
66	2014	2	2	3	3	1
67	2015	2	4	2	4	1
68	2016	1	6	2	4	1
69	2013	3	2	2	4	1
70	2014	3	5	4	1	1
71	2012	1	4	1	4	1
72	2016	2	2	2	1	1
73	2015	2	2	4	1	1
74	2013	1	2	1	4	1
75	2012	1	4	3	1	1
76	2014	2	1	3	4	1
77	2014	3	4	1	4	1
78	2014	2	3	1	3	1
79	2013	2	6	3	3	1
80	2016	1	2	1	4	1
81	2014	2	2	1	2	1
82	2015	1	2	1	1	1
83	2016	3	4	3	3	1
84	2014	3	4	2	2	1
85	2012	3	4	1	2	1
86	2015	2	2	1	2	1
87	2014	2	4	2	1	1
88	2014	3	5	2	2	1
89	2012	1	4	3	4	1
90	2016	3	2	2	1	1
91	2013	1	5	1	1	1
92	2014	3	2	1	2	1
93	2014	1	4	1	1	1
94	2013	3	6	1	3	1
95	2014	2	6	1	1	1
96	2016	2	4	3	2	1
97	2015	3	1	2	3	1
98	2013	3	3	3	1	1
99	2016	2	4	1	3	1

100	2012	3	2	2	1	1
101	2012	2	2	3	3	1
102	2013	3	4	1	1	1
103	2016	2	2	2	4	1
104	2013	1	2	1	4	1
105	2014	1	4	1	2	1
106	2012	2	4	2	3	1
107	2013	2	4	4	2	1
108	2016	3	6	1	4	1
109	2013	2	2	1	4	1
110	2014	1	3	1	3	1
111	2015	3	2	1	3	1
112	2016	2	2	1	1	1
113	2016	2	4	1	3	1
114	2016	3	4	3	3	1
115	2012	1	3	2	3	1
116	2014	3	4	1	3	1
117	2015	1	4	2	4	1
118	2016	1	4	1	2	1
119	2013	3	5	2	2	1
120	2014	1	5	3	1	1
121	2016	1	6	1	1	1
122	2014	1	2	3	2	1
123	2016	3	4	2	2	1
124	2014	1	4	1	4	1
125	2013	3	4	2	1	1
126	2012	2	4	2	4	1
127	2015	3	4	2	1	1
128	2012	2	6	2	3	1
129	2013	1	6	2	3	1
130	2013	2	5	2	2	1
131	2016	3	4	2	2	1
132	2013	2	5	1	1	1
133	2013	3	5	4	1	1
134	2012	3	5	1	1	1
135	2013	2	6	1	2	1
136	2016	2	5	1	1	1
137	2015	2	6	1	2	1
138	2014	3	4	2	1	1
139	2012	3	6	1	2	1
140	2013	1	4	1	1	1
141	2015	3	6	1	2	1
142	2012	2	4	1	4	1
143	2013	1	4	1	2	1
144	2014	2	4	3	4	1
145	2014	3	6	1	1	1
146	2015	3	5	1	1	1
147	2015	1	2	1	3	1
148	2016	1	2	1	1	1
149	2013	2	4	1	1	1

150	2012	1	2	1	2	1
151	2016	3	4	3	3	1
152	2012	1	6	1	1	1
153	2016	2	6	1	1	1
154	2013	1	2	1	3	1
155	2012	2	4	3	2	1
156	2013	1	6	3	3	1
157	2015	2	4	2	1	1
158	2016	1	4	1	3	1
159	2014	1	4	1	3	1
160	2016	1	4	4	2	1
161	2014	1	6	1	3	1
162	2015	3	4	2	4	1
163	2015	1	4	1	3	1
164	2015	1	4	2	1	1
165	2015	2	4	1	3	1
166	2012	2	6	3	2	1
167	2015	1	3	1	2	1
168	2012	2	2	1	1	1
169	2015	1	4	4	3	1
170	2012	1	2	2	3	1
171	2014	2	4	1	1	1
172	2016	1	4	4	2	1
173	2015	2	3	1	2	1
174	2014	3	6	2	2	1
175	2015	1	6	2	2	1
176	2012	2	3	2	3	1
177	2012	1	4	3	2	1
178	2014	1	4	4	1	1
179	2012	1	4	3	3	1
180	2012	1	2	2	1	1
181	2012	2	5	1	1	1
182	2013	1	6	2	2	1
183	2015	1	2	3	2	1
184	2015	3	6	4	2	1
185	2013	3	6	3	2	1
186	2015	3	4	4	3	1
187	2015	2	5	3	1	1
188	2014	1	6	4	2	1
189	2013	1	4	2	1	1
190	2016	2	4	1	2	1
191	2012	1	5	1	1	1
192	2013	2	3	1	1	1
193	2013	1	5	2	2	1
194	2014	2	5	3	1	1
195	2012	3	4	2	1	1
196	2012	3	5	4	4	1
197	2012	1	5	1	4	1
198	2012	3	2	1	2	1
199	2012	2	2	2	2	1

200	2012	3	4	1	2	1
201	2012	3	4	1	3	1
202	2012	3	4	1	4	1
203	2012	3	2	2	3	1
204	2012	1	6	2	3	1
205	2012	3	3	1	3	1
206	2013	3	2	1	3	1
207	2013	1	1	1	4	1
208	2013	2	2	1	3	1
209	2013	1	2	2	3	1
210	2013	3	4	1	2	1
211	2013	1	4	1	2	1
212	2013	3	4	1	2	1
213	2013	3	6	2	3	1
214	2013	2	6	1	3	1
215	2014	1	4	1	4	1
216	2014	2	4	2	4	1
217	2014	2	4	1	3	1
218	2014	3	3	3	3	1
219	2014	1	1	1	4	1
220	2014	3	4	1	1	1
221	2014	3	2	1	4	1
222	2015	2	6	3	4	1
223	2015	1	5	2	1	1
224	2015	3	4	2	4	1
225	2015	2	1	1	3	1
226	2015	1	4	1	4	1
227	2015	3	4	1	3	1
228	2015	2	2	1	3	1
229	2015	2	6	2	2	1
230	2015	1	6	1	2	1
231	2015	3	3	1	2	1
232	2016	1	5	1	1	1
233	2016	2	6	1	2	1
234	2016	1	1	1	4	1
235	2016	3	2	3	3	1
236	2016	1	2	1	3	1
237	2016	2	5	1	4	1
238	2016	1	5	1	4	1
239	2016	2	4	2	3	1
240	2016	3	4	3	2	1
