# Supplementary material for: Junior to senior transition of male elite junior tennis players: A retrospective study
Source: PLoS One. 2024 Oct 23;19(10):e0309472. doi: 10.1371/journal.pone.0309472 (PMC11498654; doi:10.1371/journal.pone.0309472)
Supplement: Attachment — Submitted filename: PONE-D-24-14066_reviewer MC comments.pdf [file pone.0309472.s002.pdf]

PLOS ONE

Junior to senior transition of male elite junior tennis players: a retrospective study

--Manuscript Draft--

**Manuscript Number:**	PONE-D-24-14066
**Article Type:**	Research Article
**Full Title:**	Junior to senior transition of male elite junior tennis players: a retrospective study
**Short Title:**	Junior to senior transition of male elite junior tennis players
**Corresponding Author:**	Michal Bozděch, Ph.D.<br>Masarykova Univerzita Fakulta sportovnich studii<br>Brno, Česká republika CZECH REPUBLIC
**Keywords:**	JTS; boys; male; sport; professional; talent; Association; ATP Rank
**Abstract:**	This study explores the intricate dynamics of the Junior-to-Senior (JTS) transition phase in elite tennis. Focusing on challenges faced by young talents, the research aims to unveil factors influencing successful transitions and the role of elite junior tournaments. In a retrospective analysis, male tennis players (n = 240) from national teams in the elite junior tournament (2012-2016) were analyzed using Chi-square tests, Cramer's V, Bayesian statistics, and Multinomial Logistic Regression (MLR). Results revealed 62.1% elite junior participants in the professional's association database, emphasizing the significance of team nominations and tournament results as important variable to monitoring. Inferential and Bayesian statistics confirmed robustness, with MLR highlighting tournament results' importance. The findings highlight the complexities of JTS and underscore the pivotal roles of participation, national team nominations, and tournament results. The study recommends comprehensive player development programmes, urging strategic team selections by national federations and academies. Coaches, stakeholders, and organisations should prioritise monitoring these variables for early talent identification and support. These measures collectively aim to optimise success trajectories, navigating the critical JTS phase in junior tennis players' sporting careers.
**Order of Authors:**	Michal Bozděch, Ph.D.
	Jiří Zháněl
**Additional Information:**	
**Question**	**Response**
**Financial Disclosure**<br><br>Enter a financial disclosure statement that describes the sources of funding for the work included in this submission. Review the submission guidelines for detailed requirements. View published research articles from *PLOS ONE* for specific examples.<br><br>This statement is required for submission and **will appear in the published article** if the submission is accepted. Please make sure it is accurate.	The author(s) received no specific funding for this work.

Powered by Editorial Manager® and ProduXion Manager® from Aries Systems Corporation

**Funded studies**<br>Enter a statement with the following details:<br>• Initials of the authors who received each award<br>• Grant numbers awarded to each author<br>• The full name of each funder<br>• URL of each funder website<br>• Did the sponsors or funders play any role in the study design, data collection and analysis, decision to publish, or preparation of the manuscript?<br><br>Did you receive funding for this work?	
**Competing Interests**<br><br>Use the instructions below to enter a competing interest statement for this submission. On behalf of all authors, disclose any competing interests that could be perceived to bias this work—acknowledging all financial support and any other relevant financial or non-financial competing interests.<br><br>This statement is required for submission and **will appear in the published article** if the submission is accepted. Please make sure it is accurate and that any funding sources listed in your Funding Information later in the submission form are also declared in your Financial Disclosure statement.<br><br>View published research articles from *PLOS ONE* for specific examples.	The authors have declared that no competing interests exist.

Powered by Editorial Manager® and ProduXion Manager® from Aries Systems Corporation

**NO authors have competing interests**<br><br>Enter: *The authors have declared that no competing interests exist.*<br><br>**Authors with competing interests**<br><br>Enter competing interest details beginning with this statement:<br><br>*I have read the journal's policy and the authors of this manuscript have the following competing interests: [insert competing interests here]*<br><br>* typeset	
**Ethics Statement**<br><br>Enter an ethics statement for this submission. This statement is required if the study involved:<br><br>• Human participants<br>• Human specimens or tissue<br>• Vertebrate animals or cephalopods<br>• Vertebrate embryos or tissues<br>• Field research<br><br>Write "N/A" if the submission does not require an ethics statement.<br><br>General guidance is provided below. Consult the submission guidelines for detailed instructions. **Make sure that all information entered here is included in the Methods section of the manuscript.**	The research adhered to the principles outlined in the Declaration of Helsinki and received approval from the Masaryk University Research Ethics Committee (EVK-2021-006).

**Format for specific study types**<br><br>**Human Subject Research (involving human participants and/or tissue)**<br>• Give the name of the institutional review board or ethics committee that approved the study<br>• Include the approval number and/or a statement indicating approval of this research<br>• Indicate the form of consent obtained (written/oral) or the reason that consent was not obtained (e.g. the data were analyzed anonymously)<br><br>**Animal Research (involving vertebrate animals, embryos or tissues)**<br>• Provide the name of the Institutional Animal Care and Use Committee (IACUC) or other relevant ethics board that reviewed the study protocol, and indicate whether they approved this research or granted a formal waiver of ethical approval<br>• Include an approval number if one was obtained<br>• If the study involved *non-human primates*, add *additional details* about animal welfare and steps taken to ameliorate suffering<br>• If anesthesia, euthanasia, or any kind of animal sacrifice is part of the study, include briefly which substances and/or methods were applied<br><br>**Field Research**<br><br>Include the following details if this study involves the collection of plant, animal, or other materials from a natural setting:<br>• Field permit number<br>• Name of the institution or relevant body that granted permission	
**Data Availability**<br><br>Authors are required to make all data underlying the findings described fully available, without restriction, and from the time of publication. PLOS allows rare exceptions to address legal and ethical concerns. See the PLOS Data Policy and FAQ for detailed information.	Yes - all data are fully available without restriction

A Data Availability Statement describing where the data can be found is required at submission. Your answers to this question constitute the Data Availability Statement and **will be published in the article**, if accepted.<br><br>**Important:** Stating 'data available on request from the author' is not sufficient. If your data are only available upon request, select 'No' for the first question and explain your exceptional situation in the text box.<br><br>Do the authors confirm that all data underlying the findings described in their manuscript are fully available without restriction?	
**Describe where the data may be found in full sentences. If you are copying our sample text, replace any instances of XXX with the appropriate details.**<br><br>• If the data are **held or will be held in a public repository**, include URLs, accession numbers or DOIs. If this information will only be available after acceptance, indicate this by ticking the box below. For example: *All XXX files are available from the XXX database (accession number(s) XXX, XXX.).*<br>• If the data are all contained **within the manuscript and/or Supporting Information files**, enter the following: *All relevant data are within the manuscript and its Supporting Information files.*<br>• If neither of these applies but you are able to provide **details of access elsewhere**, with or without limitations, please do so. For example:<br><br>*Data cannot be shared publicly because of [XXX]. Data are available from the XXX Institutional Data Access / Ethics Committee (contact via XXX) for researchers who meet the criteria for access to confidential data.*<br><br>*The data underlying the results presented in the study are available from (include the name of the third party*	All relevant data are within the manuscript and its Supporting Information files (S1).

and contact information or URL).<br>• This text is appropriate if the data are owned by a third party and authors do not have permission to share the data.<br><br>* typeset	
Additional data availability information:	

Manuscript Click here to access/download;Manuscript;JTS male Tennis.docx

Junior to senior transition of male elite junior tennis players: a retrospective study

Michal Bozděch[1*], Jiří Zháněl[2]

[1] Departmentof of Physical Education and Social Sciences, Faculty of Sports Studies, Masaryk University, Brno, Czech Republic

[2] Department of Sport Performance and Exercise Testing, Faculty of Sports Studies, Masaryk University, Brno, Czech Republic

*Corresponding author

E-mail: michal.bozdech@fsps.muni.cz (MB)

Abstract

This study explores the intricate dynamics of the Junior-to-Senior (JTS) transition phase in elite tennis. Focusing on challenges faced by young talents, the research aims to unveil factors influencing successful transitions and the role of elite junior tournaments. In a retrospective analysis, male tennis players (n = 240) from national teams in the elite junior tournament (2012-2016) were analyzed using Chi-square tests, Cramer's V, Bayesian statistics, and Multinomial Logistic Regression (MLR). Results revealed 62.1% elite junior participants in the professional's association database, emphasizing the significance of team nominations and tournament results as important variable to monitoring. Inferential and Bayesian statistics confirmed robustness, with MLR highlighting tournament results' importance. The findings highlight the complexities of JTS and underscore the pivotal roles of participation, national team nominations, and tournament results. The study recommends comprehensive player development programmes, urging strategic team selections by national federations and academies. Coaches, stakeholders, and organisations should prioritise monitoring these variables for early talent identification and support. These measures collectively aim to optimise success trajectories, navigating the critical JTS phase in junior tennis players' sporting careers.

which tournament?

revealed that

which professional association database?

transition

the implementation of comprehensive player…

Keywords: JTS, boys, male, sport, professional, talent, association, ATP Rank.

Introduction

may be find another word?

Tennis stands as one of the most widely embraced sports, marked by rigorous demands on physical fitness, technical prowess, and psychological resilience [1,2]. Alternatively described as a multidirectional, high-intensity intermittent individual or team racket sport, played across various surfaces by both men and women, it holds a prominent position in the athletic landscape [3,4]. Due to the prolonged development of essential skills and capabilities, the preparation for a professional sports career commences at a relatively young age.

what does this mean?

In not only tennis but also across all sports, there is an urgent need to scrutinize the transition from junior to professional categories for both genders and address issues such as Burnout, Transitions, or dual careers [2,5–7]. Due to a decline in active physical activity among adolescents [8,9], there is an increased emphasis on active and high-quality monitoring for those aspiring to maintain an active lifestyle. Therefore, alongside monitoring performance characteristics, there is a growing focus on studies that delve into the junior-to-senior transition (JST), recognized as a pivotal phase in the development of young, promising, or elite athletes [10]. This scrutiny allows for a better understanding, prediction, or reduction of Burnout or premature termination of a sports career before reaching peak performance, often associated with a disdain for physical activity as well [11,12]. The JST phase is crucial in the development of young, promising, or elite athletes, signifying the shift from junior/youth age categories to the professional level [13]. Typically lasting between one and four years, this phase is characterized by non-linear processes, presenting a dynamic and complex developmental stage accompanied by sociocultural barriers and heightened expectations [14–16]. Athletes navigate this transitional phase amidst elevated psychological stress, emotional, moral, and performance-related standards, making it the most challenging and critical period in their sporting careers [13,17,18].

why in capital?

Elite players strive to overcome it with the least risk of injury and negative consequences [13,17–19]. Consequently, the dropout rate among junior athletes during this phase can be as high as two-thirds [20]. While temporary biological advantages related to birth dates may aid relatively older peers, evidence indicates a higher dropout rate among those who unknowingly benefited from this advantage, particularly after adolescence and within two years at the professional level; often before reaching their peak performance evident [12,21–24].

The complexity of tennis, akin to most high-performance sports, involves multifaceted associations and covariations impacting the course and outcome of the game, tournament, season, health, JTS transition, and the entire sports career. These associations encompass performance-based as well as socio-cultural, financial, technological, and stakeholder support elements [25,26]. The objectives of this study resulted from this, i.e., to analyze the probability of elite junior tennis players entering professional tennis association. Therefore, the objective of this

retrospective study is to develop a valid model for predicting whether participants in elite junior tournaments will become professional tennis players.

It seems that the text included in this introduction is quite general. it would be convenient to identify in the introduction the main aspects of JTS transition in tennis and, as a consequence of the research gaps identified in the introduction, put forward the goals of the study.

Methods

Participants

Tennis players from national representative teams who advanced to The World Junior Tennis Final (WJTF) tournament between 2012 and 2016 were participants in this retrospective-predictive study. This time frame was intentionally selected to allow participants the opportunity to reach their peak performance age (around 24 years) during the data collection year [27,28]. To ensure study accuracy and minimize potential gender-related influences [19], the research exclusively focused on male tennis players. Out of the total 240 tracked players, eight participated in the elite tournament more than once. It is important to note that the results of the pilot study were presented at a conference. However, different statistical methods were applied for this research to confirm a different research intent and objectives. The research adhered to the principles outlined in the Declaration of Helsinki and received approval from the Masaryk University Research Ethics Committee (EVK-2021-006). Even if the data was anonymized after completion. Due to the design and objectives of this study, the authors were able to identify individual participants. However, due to the retrospective nature of this research, this did not affect the results, but served to provide a more detailed understanding of the investigated effects.

It would be convenient to provide the main characteristics that emphasise the importance of this tournament

Data collection

Researchers obtained foundational data and consent to use it from tournament organizers, consisting of official materials, for each year of the elite tournament. Subsequently, these materials were supplemented with information related to the sports career, acquired from the official publicly accessible Association of Tennis Professionals (ATP) website

(https://www.atptour.com/en/). Specifically, players were individually searched and classified by ATP status in single; Not Found (NF), Registered (R) without points, and Found (F). For Found tennis players who accumulated sufficient points from ATP tournaments, the value of their best career ranking was also obtained (updated as of the last week of the 2022 season; data collection from the professional association took place retrospectively in the first quarter of 2023). Data were validated and cleaned of outliers.

Research criteria

To address the research objectives, the data was categorized according to the following criteria: *ATP Status (F/NF)* [dichotomous data, Found or Not Found in ATP database]; *ATP Status (F/R/NF)* [nominal data, Registered without points, Found, and Not Found in ATP database; *Nomination* [ordinal data, 1-3 rank, a team in an elite junior tournament consists of 3 tennis players with 3 predefined levels, often determined according to the current national ranking]; *Year of the tournament* [nominal data; 2012-2016 year, five consecutive tournaments from 2012 through 2016]; *Continent* [nominal data, Countries represented by individual tennis teams were converted into continents based on their predominant geographical location]; *Birth quarter* [nominal data, Q_1-Q_4, obtained from the birth date, precisely the month, which was then converted into quarters, January-March, April-June, etc.]; *WJTF results* [ordinal data, 1-4 category, each year, 16 teams participated in the tournament, data was converted into quarters, 1st-4th place, 5th-8th place, etc.].

Statistical analysis

The statistical analysis chosen to address the research objectives initially included methods and procedures from inferential statistics (Chi-square; χ^2), Effect Size (Cramer's V), Bayesian approach, Regression analysis (Multinomial logistic regression, MLR).

This approach was chosen to support complementarity between Inferential statistics, Effect Size, and the Bayesian approach [29]. Chi-square (χ^2) test of independence, Cramer's V

test, with Cohen (1988) interpretation, and Bayesian statistics (BF_{10} and BF_{01}) were utilized to evaluate the different frequencies among participants in the elite tournament who were Found or Not Found in the ATP database, depending on various research variables (Nomination, Tournament year, Continent, Birth quartile, WJTF results). A priori Power analysis for the Chi-square test was conducted considering the expected effect size, level of significance, and test power, utilizing G*Power 3.1.9.6 software. For a medium effect, with α = 0.05 and 1-β = 0.95, a total sample of 172 participants was required, based on the values derived from the WJTF results variable. With the current participant count in the study (*n* = 240), at α = 0.05, the Power (1-β) exceeds 0.99. The most prominent variables from this phase (Nomination and WJTF results) were further analyzed using MLR, where the dependent variable was ATP status (the reference category being Not Found in the ATP database), and the factors were Nomination and WJTF results. Out of 240 observations, no data were missing. The Likelihood Ratio chi-square test was used to compare the full model against a null or no predictors model. The final model showed a significant improvement in fit over a null model; $\chi^2(5) = 11.68$, $p = .039$, indicating that the full model represents a significant improvement in fit over the null model. The Goodness of Fit test indicated that both Pearson's [$\chi^2(6) = 4.79$, $p = .571$] and Deviance [$\chi^2(6) = 5.01$, $p = .543$] Chi-square tests indicated a good fit. The statistical analysis was conducted using IBM SPSS Statistics (Version 29.0.0, IBM Corp, Armonk, NY) and JASP (Version 0.18.3, University of Amsterdam) software packages for Windows. Statistical significance was assessed using a significance level (α) of 0.05. Therefore, results with a p-value less than 0.05 were considered statistically significant.

Results

Among the 240 participants in the elite junior tournament, 195 were identified in the official ATP database. However, 46 were merely registered and did not acquire any points, while 149 (62.08%) earned official ranking points. Of these 149 players, 64 (42.95%) achieved a position within the top 500 in their tennis careers, 40 (26.85%) achieved places in the 501-1000 rank, 29

(19.46%) achieved in the 1001-1500 rank category, and 16 (10.74%) attained rankings of 15001 and above. This suggests a negative association between player frequency and their best career rankings. In other words, participants in the elite tournament more frequently achieved higher career ranking. Upon a closer examination within 100-rank intervals, it was observed that the majority (n = 18) of elite tournament participants reached the 201-300 rank, constituting 7.50% of the entire research sample and 12.08% from those who earns enough ATP points for ranking.

Table 1. An Overview of ATP Status Distribution according to Research Variables.

**Variable**		**Players ATP status**, *n* (%)		***n***	***p***	**V**	**BF_{10}**	**BF_{01}**
		**Not found**	**Found**					
Nominations in team	1st	25 (10.42 %)	55 (22.92 %)	80	.024	.176*	1.58	0.63
	2nd	26 (10.83 %)	54 (22.50 %)	80				
	3rd	40 (16.67 %)	40 (16.67 %)	80				
Year of the tournament	2012	19 (15.83 %)	29 (24.17 %)	48	.435	.126*	0.03	32.83
	2013	14 (11.67 %)	34 (28.33 %)	48				
	2014	17 (14.17 %)	31 (25.83 %)	48				
	2015	18 (15.00 %)	30 (25.00 %)	48				
	2016	23 (19.17 %)	25 (20.83 %)	48				
Continent	Africa	12 (13.19 %)	6 (6.59 %)	18	.070	.206**	0.15	6.51
	Asia	22 (24.18 %)	29 (31.87 %)	51				
	Australia	4 (4.40 %)	8 (8.79 %)	12				
	Europe	24 (26.37 %)	60 (65.93 %)	84				
	N. America	12 (13.19 %)	18 (19.78 %)	30				
	S. America	17 (18.68 %)	28 (30.77 %)	45				
Birth quarter	Q_1	52 (21.67 %)	72 (30.00 %)	124	.312	.122*	0.04	22.66
	Q_2	25 (10.42 %)	39 (16.25 %)	64				
	Q_3	9 (3.75 %)	25 (10.42 %)	34				
	Q_4	5 (2.08 %)	13 (5.42 %)	18				
WJTF results	1.-4. place	13 (5.42 %)	47 (19.58 %)	60	.002	.251**	24.5	0.04
	5.-8. place	20 (8.33 %)	40 (16.67 %)	60				
	9.-12. place	25 (10.42 %)	35 (14.58 %)	60				
	13.-16. place	33 (13.75 %)	27 (11.25 %)	60				
Total		91 (37.92 %)	149 (62.08 %)	240				

Q_i birth quarter. *small ES. **Medium ES. ***Large ES. BF_{10} Bayes factor in favor alternative hypothesis over null hypothesis. BF_{01} Bayes factor in favor null hypothesis over alternative hypothesis.

The Chi-square test revealed significant differences in the proportions of Players ATP status between various levels of Nominations in the team ($\chi^2(2) = 7.47$, $p = .024$) and WJTF results (place) ($\chi^2(3) = 15.06$, $p = .002$). However, after Bonferroni correction ($p_{adj} = .001$), only WJTF results remained statistically significant. Based on descriptive statistics, this suggests that a better Nomination in the team indicates a higher likelihood of being registered (and consequently entering) into the professional senior category. A similar observation can be made

regarding the final ranking of the team in the elite tournament, where this effect was more pronounced, as confirmed by the conclusions drawn from Effect Size (ES) and Bayes Factor (BF). Specifically, it can be asserted that the better the team's position, the higher the probability of reaching the ATP, with a reverse effect for teams placed at the bottom of the table. As the variables listed in Table 1 had different levels and, consequently, different degrees of freedom, the results of the substantive significance test will be presented using verbal classification: trivial, small, medium, large. In this study, a Small to Medium effect was identified, specifically 3× small and 2× medium effects. The medium effect is noticeable for variables that were statistically significant (based on the χ^2 test), indicating a moderate association between Players ATP status and Nomination in the team or results at the elite junior tennis tournament.

However, based on the results of Bayes statistics presented in Table 1, those two statistically significant variables with moderate associated fields are nearly 1.6 and 24.5 times more likely to have differences between variables than not, providing a more accurate quantification of the differential influence between these variables. Not only according to Bayesian statistics but also based on other observations, it can be argued that not only the level of nomination in the team but especially the team's final ranking at the junior tournament significantly influences the future sports career trajectory of young and promising tennis players.

Figure 1. An Overview of Association between Nomination in Team and tournament Results.

In Fig 1, a smooth regression line with a 95% confidence interval has been incorporated for a more precise depiction of errors. The interval appears narrower in the 'Found' category compared to the 'Not Found' category, indicating higher precision. Fig 1 visually demonstrates the effect alluded to in the previous paragraph – specifically, a lesser nomination in the elite team, leading to lower performance in the junior tournament, heightens the probability of terminating a tennis career in sports before reaching peak performance and even before the career fully commences.

Building on the aforementioned findings, the MLR method was employed. In this analysis, the dependent variable was ATP status, with the reference category being 'Not Found' in the ATP database, and the factors considered were Nomination and WJTF results, both treated as ordinal variables. Results from the Likelihood Ratio test indicate that the factor WJTF results (p = .041) was a statistically significant predictor in the model and, therefore, contributed more significantly to the overall variance. On the other hand, the factor Nomination was not statistically significant (p = 0.17).

The regression coefficients reveal that predictors in WJTF results (1st to 3rd level) significantly discriminate between players who were not found in the ATP database and those who were found. Specifically, players who achieved 1st to 4th place (β = 0.983, SE = 0.460, p = .033), 5th to 8th place (β = 0.983, SE = 0.460, p = .033), and 9th to 12th place (β = 1.122, SE = 0.474, p = .018) had significant coefficients. The Odds Ratio was 2.67 for 1st to 4th place (95% CI = 1.085, 6.580), 2.672 for 5th to 8th place (95% CI = 1.085, 6.580), and 3.070 for 9th to 12th place (95% CI = 1.211, 7.780). The positive β coefficient in these variables indicates that players who achieved a worse place (higher order number) had a higher chance of being found in the ATP database. The odds ratio suggests that for every one-unit increase in WJTF results (e.g., worse results), the odds of a player being found in the ATP database change by a factor of 2.7 to 3.1, indicating an increasing likelihood.

This reaffirms the earlier findings that WJTF results are the most crucial factor among the observed variables. However, it extends these conclusions by providing additional information: it is not the teams that achieved 1st-4th place at the tournament that have the highest likelihood of being identified, but rather participants from teams ranking 9th-12th place who have the greatest odds. Nevertheless, the differences are not as pronounced. do you mean significant?

Discussion

The present study…

Study underscores the formidable challenges inherent in the transition from elite junior tennis to the ATP, emphasizing the pivotal role of the JST phase. To enhance the prospects of

aspiring players, national tennis federations and academies should implement comprehensive player development programs that prioritize both individual performance and strategic team dynamics. The findings highlight the importance of participating in an elite junior tournament (through an unexpectedly high percentage of junior elite tennis players making it to the professional association); the significance of strategic team nominations (demonstrating that teams with higher nominations, even if not securing victory, exhibit a heightened likelihood of successful transitions to the ATP) and relevance of results at tournament, which were evident in descriptive statistics (the relative number of tennis players who made it to the professional association increased with a better team position, but in the case of those who did not make it to the ATP, on the contrary, it decreased with a worse position) and confirmed using inferential statistics (χ^2, ES, BF), but a similar trend was not demonstrated in the case of MLR, where (albeit negligibly) players with a worse team position have a greater chance of getting into the ATP. These discrepancies in conclusions are most likely the result of applying different statistical methods that answer different questions in different ways.

achieving a professional ranking

at that tournament or alt all tournaments?

the descriptive

Moreover, a similar study focusing on elite junior female tennis players transitioning to the Women's Tennis Association (WTA) found that out of 240 participants in an elite junior tournament, 141 (58.8%) gained points in the WTA rank [25]. Eight participants less (3.3%) than reported in the current study (*n* = 149, 62.1%). Both studies revealed a lower dropout rate (girls = 41.3%, boys = 37.9%) than reported by Franck et al. (2018); up to 75.0% dropout rate among junior athletes. Suggesting that participation in elite junior tournaments plays a crucial role in JTS transition. However, it is essential to note that other important factors were not examined within the scope of this study, such as skills, psychological factors, health status, socio-cultural and economic factors, among others.

This is pretty obvious!

The study indicates that participants in elite junior tournaments commonly achieve rankings in the 201-300 range in their best career ATP ranking. This aligns with Balliauw et al. [31] assertion that professional tennis players ranked worse than the top 250 face financial challenges, potentially influencing the continuation of their sports careers. Therefore, we could

state that this is an important professional threshold placement and coaches, players, associations and stakeholders should be aware of that.

Limitations and directions for future research

Despite the diligent efforts of researchers, this study has its limitations. The focus on participants in elite competitions may overlook valuable information about those who did not reach the final but performed well in semi-finals or at the state level, just below the team selection threshold. Expanding the dataset could enhance the understanding of the JTS process, but they would not add new data on tournament nominations and results from that elite junior tournament, which turned out to be the most important variables.

The potential limitation can be addressed by acknowledging that our primary data, obtained from an elite junior tennis tournament, were combined with information from a secondary source (official ATP website). However, this is not a unique approach to data acquisition and analysis, as indicated by an extensive study by Takahashi et al. [32], who identified the existence of six common methods for obtaining tennis data (data mining, tracking, video recording, coaches, broadcasting, and Internet). Out of 90 different studies, 38 studies utilized a similar approach to data acquisition (secondary data from publicly available web sources). Therefore, this represents a standard practice in the specific sports environment. Nevertheless, for determining causal relationships, it would be advisable to conduct a longitudinal study, preferably in collaboration with sports associations, coaches, stakeholders, and other researchers.

the official

The current study exclusively focused on male tennis players, excluding their female counterparts due to substantial disparities observed in the performance, strategy. These distinctions are not only evident across the spectrum of professional male and female players but also between junior and professional male players [33–36]. This emphasizes the need for gender-specific as well as junior and senior-specific training, highlighting the imperative adoption of distinct training methods and the customization of approaches based on performance levels. It

also underscores the essential development of suitable methods tailored for monitoring in each respective sex and phase.

Conclusion

This study delves into the challenges of transitioning from elite junior to professional tennis, known as the JST Transition, emphasizing, heightened expectations, sociocultural barriers, and psychological stress. Key findings highlight the predictive power of participation in the Elite Junior Tennis Tournament, as 62.1% elite junior participants progressed to actively participate in the ATP.

Better rankings at the elite junior tournament associated with higher chances of being present in the ATP ranking database and having the possibility of successful professional careers. Team nomination at the elite junior tournament, but especially results at this tournament, are crucial predictors for future professional status. Although multiple linear regression confirms the importance of elite tournament placement, in-depth analysis does not show a significant advantage for higher-ranked players or teams in reaching the professional association.

To enhance support for athletes during the challenging JST phase, it is advisable to establish a robust longitudinal monitoring system. Collaborative efforts among tennis organizations, coaches, stakeholders, and sports scientists can facilitate comprehensive tracking of players' progress, enabling timely interventions and support. Additionally, the implementation of educational programs addressing the unique stressors and challenges associated with JST is crucial. Coaches, scouts, and tennis organizations should prioritize monitoring elite junior tournament results for early talent identification. Overall, a holistic approach to player development, strategic team selections, and the judicious use of predictive analytics are recommended for optimizing the success trajectories of junior tennis players in their professional sport journeys.

Reference

1. Lambrich J, Muehlbauer T. Effects of athletic training on physical fitness and stroke velocity in healthy youth and adult tennis players: A systematic review and meta-analysis. Front Sports Act Living. 2023;4. doi:10.3389/fspor.2022.1061087

2. Kovacs MS. Tennis physiology: training the competitive athlete. Sports Medicine. 2007;37: 189–198. doi:0112-1642/07/0003-0189/$44.95/0

3. Pluim BM, Jansen MGT, Williamson S, Berry C, Camporesi S, Fagher K, et al. Physical Demands of Tennis Across the Different Court Surfaces, Performance Levels and Sexes: A Systematic Review with Meta-analysis. Sports medicine. 2023. doi:10.1007/s40279-022-01807-8

4. Reilly T, Morris T, Whyte G. The specificity of training prescription and physiological assessment: A review. J Sports Sci. 2009;27: 575–589. doi:10.1080/02640410902729741

5. Devine JW. Elements of excellence. J Philos Sport. 2022;49: 195–211. doi:10.1080/00948705.2022.2059489

6. Lambrich J, Muehlbauer T. Physical fitness and stroke performance in healthy tennis players with different competition levels: A systematic review and meta-analysis. PLoS One. 2022;17: e0269516. doi:10.1371/journal.pone.0269516

7. Tessitore A, Capranica L, Pesce C, De Bois N, Gjaka M, Warrington G, et al. Parents about parenting dual career athletes: A systematic literature review. Psychol Sport Exerc. 2021;53: 101833. doi:10.1016/j.psychsport.2020.101833

8. Hallal PC, Victora CG, Azevedo MR, Wells JCK. Adolescent Physical Activity and Health A Systematic Review. Sports Med. 2006;36: 1019–1030. doi:0112-1642/06/0012-1019/$39.95/0

9. van Sluijs EMF, Ekelund U, Crochemore-Silva I, Guthold R, Ha A, Lubans D, et al. Physical activity behaviours in adolescence: current evidence and opportunities for intervention. The Lancet. 2021;398: 429–442. doi:10.1016/S0140-6736(21)01259-9

10. Drew K, Morris R, Tod D, Eubank M. A meta-study of qualitative research on the junior-to-senior transition in sport. Psychol Sport Exerc. 2019;45. doi:10.1016/j.psychsport.2019.101556

11. Alfermann D, Stambulova N. Career Transitions and Career Termination. In: Tenenbaum G, Eklund RC, editors. Handbook of Sport Psychology. Wiley; 2007. pp. 712–733. doi:10.1002/9781118270011.ch32

12. Alfermann D. Drop-out. In: Eklund RC, Tenenbaum G, editors. Encyclopedia of sport and exercise psychology. Sage Publications.; 2014. pp. 215–216.

13. Røynesdal Ø, Toering T, Gustafsson H. Understanding players' transition from youth to senior professional football environments: A coach perspective. Int J Sports Sci Coach. 2018;13: 26–37. doi:10.1177/1747954117746497

14. Finn J, Mckenna J, Stambulova N. Coping with Academy-to-First-Team Transitions in Elite English Male Team Sports: The Coaches' Perspective. Int J Sports Sci Coach. 2010.

15. Mills A, Butt J, Maynard I, Harwood C. Identifying factors perceived to influence the development of elite youth football academy players. J Sports Sci. 2012;30: 1593–1604. doi:10.1080/02640414.2012.710753

16. Storm LK, Kristoffer H, Krogh MC. Specialization_pathways_among_Danish_eli. Int J Sport Psychol. 2012;43: 199–222.

17. Roderick M. Adding insult to injury: workplace injury in English professional football. Sociol Health Illn. 2006;28: 76–97. doi:10.1111/j.1467-9566.2005.00483.x

18. Stambulova NB. Crisis-transitions in athletes: current emphases on cognitive and contextual factors. Curr Opin Psychol. 2017;16: 62–66. doi:10.1016/j.copsyc.2017.04.013

19. Stambulova NB. Talent Development in Sport: A Career Transitions Perspective. In: E. Tsung-Min Hung E, Lidor R, Hackfort D, editors. Psychology of Sport Excellence . Morgantown: Fitness Information Technology; 2009. pp. 63–74.

20. Franck A, Stambulova NB, Ivarsson A. Swedish athletes' adjustment patterns in the junior-to-senior transition. Int J Sport Exerc Psychol. 2018;16: 398–414. doi:10.1080/1612197X.2016.1256339

21. Fumarco L, Gibbs BG, Jarvis JA, Rossi G. The relative age effect reversal among the National Hockey League elite. PLoS One. 2017;12. doi:10.1371/journal.pone.0182827

22. Steingröver C, Wattie N, Baker J, Schorer J. Does Relative Age Affect Career Length in North American Professional Sports? Sports Med Open. 2016;2. doi:10.1186/s40798-016-0042-3

23. Crane J, Temple V. A systematic review of dropout from organized sport among children and youth. Eur Phy Educ Rev. 2015;21: 114–131. doi:10.1177/1356336X14555294

24. Delorme N, Chalabaev A, Raspaud M. Relative age is associated with sport dropout: Evidence from youth categories of French basketball. Scand J Med Sci Sports. 2011;21: 120–128. doi:10.1111/j.1600-0838.2009.01060.x

25. Bozděch M, Zháněl J. Analyzing game statistics and career trajectories of female elite junior tennis players: A machine learning approach. PLoS One. 2023;18. doi:10.1371/journal.pone.0295075

26. Park S, Lavallee D, Tod D. Athletes' career transition out of sport: a systematic review. Int Rev Sport Exerc Psychol. 2013;6: 22–53. doi:10.1080/1750984X.2012.687053

27. Schulz R, Curnow C. Peak Performance and Age Among Superathletes Track and Field, Swimming, Baseball, Tennis, and Golf. Journal olGcroiiioh. 1988;43: 113–120. Available: https://academic.oup.com/geronj/article/43/5/P113/734937

28. Allen S V., Hopkins WG. Age of Peak Competitive Performance of Elite Athletes: A Systematic Review. Sports Medicine. Springer International Publishing; 2015. pp. 1431–1441. doi:10.1007/s40279-015-0354-3

29. Quintana DS, Williams DR. Bayesian alternatives for common null-hypothesis significance tests in psychiatry: a non-technical guide using JASP. BMC Psychiatry. 2018;18: 178. doi:10.1186/s12888-018-1761-4

30. Cohen J. Statistical power analysis for the behavioral sciences. 2nd ed. Hillsdale: Lawrence Erlbaum Associates; 1988.

31. Balliauw M, Verlinden T, Van Den Spiegel T, Van Hecke J. How to achieve a sustainable circuit for professional tennis players? International Journal of Sport Management and Marketing. 2023;23: 391–418. doi:10.1504%2FIJSMM.2023.133162

32. Takahashi H, Mitsuhashi S, Murakami S. Performance analysis in tennis since 2000: A systematic review focused on the methods of data collection. International Journal of Racket Sports Science. 2022;4: 40–55. doi:10.30827/Digibug

33. Reid M, Morgan S, Whiteside D. Matchplay characteristics of Grand Slam tennis: implications for training and conditioning. J Sports Sci. 2016;34: 1791–1798. doi:10.1080/02640414.2016.1139161

34. Cui Y, Gómez M-Á, Gonçalves B, Sampaio J. Performance profiles of professional female tennis players in grand slams. PLoS One. 2018;13: e0200591. doi:10.1371/journal.pone.0200591

35. Pereira TJC, van Emmerik REA, Misuta MS, Barros RML, Moura FA. Interpersonal coordination analysis of tennis players from different levels during official matches. J Biomech. 2018;67: 106–113. doi:10.1016/j.jbiomech.2017.11.036

36. Kovalchik SA, Reid M. Comparing Matchplay Characteristics and Physical Demands of Junior and Professional Tennis Athletes in the Era of Big Data. J Sports Sci Med. 2017;16: 489–497.

Supporting information

S1 File. S1_File_Raw Data_ Male JTS Tenis.pdf.

Figure

Click here to access/download;Figure;FIGURE~1.TIF

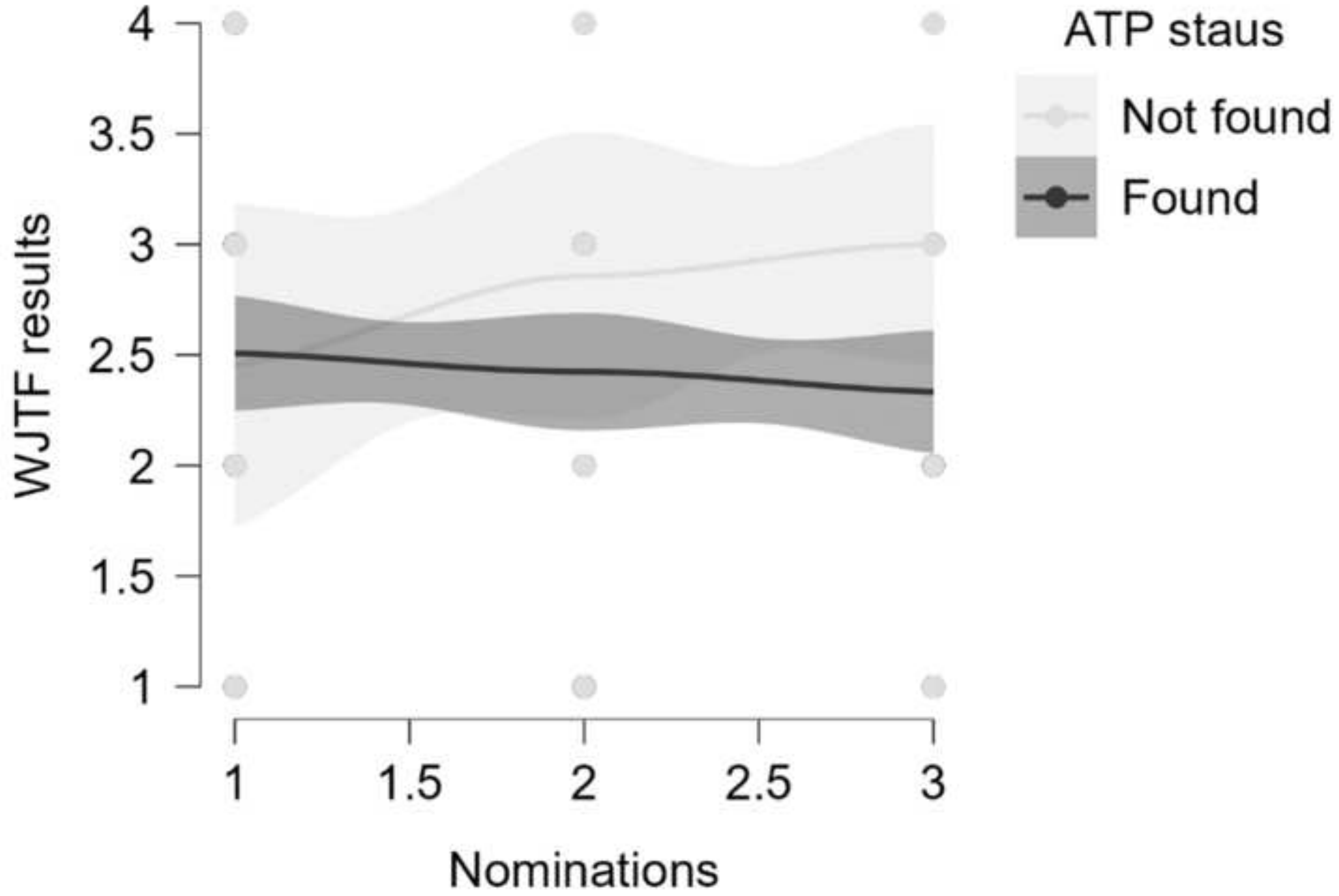

Click here to access/download
Supporting Information
S1_File_Raw Data_ JTS Male Tenis.pdf
