# Supplementary material for: Junior to senior transition of male elite junior tennis players: A retrospective study
Source: PLoS One. 2024 Oct 23;19(10):e0309472. doi: 10.1371/journal.pone.0309472 (PMC11498654; doi:10.1371/journal.pone.0309472)
Supplement: Attachment — Submitted filename: Response to Reviewers.docx [file pone.0309472.s003.docx]

**Junior to senior transition of male elite junior tennis players: a retrospective study**

**Reviewer #1**

Thank you for the opportunity to review your manuscript. At present, I do not feel the results support some of the conclusions in the discussion of the paper. In particular, I struggled to understand how the reported findings related to psychosocial stressors and pressures during the junior to senior transition.

We agree that this factor was not directly addressed in our study. Although it was mentioned to provide context for the JTS transition, for clarity and coherence, we have removed references to this factor from various parts of the manuscript, especially in the conclusions.

I also felt the varied statistics used detracted from the results, as this created some confusion when reading the manuscript and made it difficult to know which results were most important for my interpretation as a reader.

We agree that the use of multiple statistical techniques may have compromised the clarity of the manuscript. We have revised the text, removing detailed descriptions of less relevant conclusions from various statistical methods and simplifying the interpretation of results.

I also wish to note a few issues with the language throughout, including unclear language and some words that were capitalised and did not need to be, which require revisions.

We have conducted a thorough proofreading focused on correcting capitalization (e.g., burnout, transition). We are grateful for this comment, as it helped us identify other errors in our manuscript.

We believe that your review and the time you dedicated to it have significantly improved the quality of our contribution, for which we are very thankful.

**Reviewer #2**

The reviewer would like to thank the authors for doing research in such an interesting field for the sport of tennis. The following comments and questions are made with the intention of assisting the authors in improving the quality of the manuscript which is already quite high. Therefore, the reviewer would like the authors to understand these comments as part of the scientific debate that is needed for the advancement of science.

We thank you for your valuable time dedicated to reviewing our manuscript and for the thoughtful comments provided. Your feedback is absolutely valid, and we appreciate it greatly. We believe it has significantly contributed to the improvement of the entire manuscript.

To facilitate this process, the reviewer has added the pdf file with highlighted sections, comments, and questions for the authors to address.

Based on your comments, we have incorporated the following into the revised manuscript:

- **Abstract:**
  - Specified the tournament: "In a retrospective analysis, male tennis players (n = 240) from national teams in the **ITF World Junior Tennis Finals** tournament (2012-2016)..."
  - Specified the professional database: "...the Association of Tennis Professionals database..."
- Corrected minor grammatical errors, missing words for clarity, adjusted articles [a, the], used more commonly accepted synonyms, and corrected capitalization (burnout, transitions).
- Removed the section "Alternatively described as a multidirectional, high-intensity, intermittent, individual or team racket sport, played across various surfaces by both men and women, it holds a prominent position in the athletic landscape [3,4]," as it did not add new information to the introduction relevant to the study.

The main aspects of JTS transition in tennis and, as a consequence of the research gaps identified in the introduction, put forward the goals of the study

To the introduction, we added new information about the JTS in tennis, its consequences, and the research gaps. Thank you for this suggestion.

It would be convenient to provide the main characteristic that emphasise the importance of this tournament.

We agree, and we have added to the methods section: "The World Junior Tennis Finals (WJTF) is a prestigious team tournament for players aged 14 and under. The tournament has been organized by the International Tennis Federation (ITF) since 1991 and, since 1999, in collaboration with the Czech Tennis Association and the tennis club TK PLUS in Prostějov. This competition gathers the top junior boys and girls from around the world, whose teams qualify based on results from regional tournaments, offering them a competitive platform that prepares them for a professional career. Many participants in this tournament have historically transitioned to successful professional careers, including notable players such as Rafael Nadal, Novak Djokovic, Carlos Alcaraz, and Tomáš Berdych. The tournament provides young athletes with invaluable experience and opportunities to showcase their skills on a global stage. This underscores the significance of the WJTF in our study, as it serves as an ideal setting to analyze the potential of elite junior players advancing to professional levels.”

I also felt the varied statistics used detracted from the results, as this created some confusion when reading the manuscript and made it difficult to know which results were most important for my interpretation as a reader. Simplify the statistical analyses to enhance readability and focus on the most critical results that support your conclusions

We simplified the results section to make it more readable for the audience. The text is now clearer, with unnecessary details removed. This also helped us ensure that different statistical methods applied in this paper support the same results, main conclusion of the study, except for the MLR analysis, which suggests opposite and less logical conclusions. For ethical reasons, we had to mention this contradictory conclusion in the text but labeled it as unreliable and in need of further investigation. Thank you for this comment.

The reviewer believes that if the authors address these comments and make minor changes to the manuscript, it should be accepted for publication. Finally, the reviewer suggests that the authors seek for the assistance of a native English speaker person to improve some sections of the manuscript.

The manuscript underwent a language review, which revealed several grammatical and phrasing errors. We thank you for your valuable time dedicated to our article. All your comments have been incorporated into the manuscript, and we believe they have significantly enhanced the quality of this manuscript.

**Editor**

The reviewers have raised several critical points that need to be addressed to improve the clarity, coherence, and overall quality of your paper. The main issues highlighted include the clarity and relevance of the conclusions drawn from the results, the complexity and variety of statistical analyses used, and several language-related problems, including unclear wording and unnecessary capitalization. Additionally, there are concerns regarding the methodological approach and the exclusion of female players from the study, which need to be thoroughly justified.

To address these concerns, please focus on the following key areas in your revision. Firstly, ensure that the results and conclusions are directly aligned, and clarify how your findings relate to psychosocial stressors and pressures during the junior to senior transition in tennis. Simplify the statistical analyses to enhance readability and focus on the most critical results that support your conclusions. Additionally, review the manuscript for language clarity, correct any unclear wording, and remove any unnecessary capitalization. Justify the rationale for excluding female players and consider discussing the implications of this choice. Lastly, please incorporate the specific comments and questions highlighted in the attached PDF from the reviewer and seek the assistance of a native English speaker to polish the language.

Thanks for the summary and additional comments. In addition to the corrections, we've made above, we've also expanded the "Participants" section to provide a more detailed rationale for focusing on male players only:

Original text: To ensure study accuracy and minimize potential gender-related influences [19], the research exclusively focused on male tennis players.

Edited and expanded text: The research exclusively focused on male tennis players to ensure study accuracy and minimize potential gender-related influences. Previous studies have identified significant gender-related differences in performance-related variables [19]. Including both male and female players could introduce heterogeneity in the data, potentially confounding the results and leading to less reliable conclusions. By focusing solely on male players, the study aims to provide a clearer understanding of the factors influencing performance within a more homogeneous group.

We note that the grant information you provided in the ‘Funding Information’ and ‘Financial Disclosure’ sections do not match.

Thanks to the editor for noticing this deficiency. We have corrected it.
